# Specialty services offered by pharmacists in the community

**DOI:** 10.1186/s13584-019-0325-5

**Published:** 2019-07-12

**Authors:** Anthony P. Morreale, Julie A. Groppi, Heather Ourth

**Affiliations:** Department of Veterans Affairs, Clinical Pharmacy Practice Office, Pharmacy Benefits Management (PBM) Services, Washington D.C, USA

**Keywords:** Clinical services, Clinical pharmacy, Pharmacist, Scope of practice

## Abstract

In a recent IJHPR article, Schwartzberg and colleagues report on clinical and other specialty services offered by pharmacists in the community in Israel and in the international arena. The article covers examples of activities recently introduced due to legislative changes which expanded the pharmacist’s scope of practice, along with obstacles that are serving to slow broader expansion and availability of these services. This commentary details the success of clinical pharmacy services being provided by the United States Veterans Health Administration, and offers a framework of elements that support clinical pharmacy practice expansion.

## United States experience

In the United States, clinical pharmacy practice has evolved moving from a profession focused on drug products to one focused on the delivery of patient care. This transition was solidified in 2000, when new standards developed by the Accreditation Council for Pharmacy Education (ACPE) went into effect and the Doctor of Pharmacy (Pharm.D.) degree became the entry-level degree for the profession. Along with this change, there has been an increased emphasis on the need for postgraduate pharmacy residency training and board certification for clinical pharmacists (CPs) engaged in direct patient care. The evolution continues today with the push to have pharmacists and pharmacists’ patient care services recognized with provider status in the United States Social Security Act, which determines eligibility for health care programs such as Medicare Part B. Several studies, including meta-analyses, indicate CPs deliver equivalent, or in some cases (e.g., anticoagulation) superior, medication management services to patients when compared to other types of healthcare providers [1–6]. Furthermore, in the United States, 94% of jurisdictions grant pharmacists prescriptive authority in the form of collaborative drug therapy management and authorize pharmacist prescriptive authority as outlined in their individual pharmacy practice acts [7].

### Veterans health administration (VHA) experience

In the Veterans Health Administration (VHA) CPs have been autonomously and collaboratively providing complex medication management activities for over 40 years [7, 8].

The VHA is, without a doubt, one of the premier examples of what can be done with well-trained CPs serving as advanced practice providers (APP). In the VHA, CPs deliver their care under policy authority known as a Scope of Practice (SOP) which reflects an authorization to perform as an APP with the ability to autonomously and collaboratively manage all facets of a patient’s disease, condition, and medications [9]. There are approximately 8800 CPs in the VHA, of which nearly 46% are credentialed with prescriptive authority outlined in their SOP. From 2011 to the present, the number of CPs working under an SOP has risen an astonishing 142%, primarily due to focused promotion of their role in medication and disease management and their ability to improve access to needed healthcare, especially in rural areas [10]. These CPs function in a variety of practice settings including ambulatory, acute, and residential care settings, treating a vast array of conditions. Utilizing their SOP, CPs provided over 6 million patient care visits annually in Fiscal Year 2018 and play a key role in improving access to medication management services and improve the quality of care to our Veterans.

The CP SOP is focused to cover the practice area where the CP provides Comprehensive Medication Management (CMM) services, rather than being focused on disease states that they manage or medications they may prescribe. In addition, the SOP does not only include prescriptive authority but also core elements essential to the provision of CMM services, such as ordering related laboratory tests and diagnostic studies, performing physical measurements and objective assessments, making referrals for additional patient care needs, and performing other necessary activities to facilitate patient care [9]. It is imperative that these core elements are not uncoupled to ensure that the CP is able to provide a full range of services to support the provision of CMM. Within this SOP, the CP functions with a high level of autonomy and engages in independent clinical decision-making, assuming accountability for their provision of care. An example of this comprehensive SOP is seen in the primary care practice setting, where a “global” SOP encompasses chronic disease state management including, but not limited to, diabetes, hypertension, hyperlipidemia, smoking cessation, pain management, hepatitis C, and osteoporosis. Essential to the process is that each CP, based on demonstrated areas of competence, is granted the right by the individual facility to practice autonomously in those areas. This process mirrors that used for physicians with oversight by the medical staff. This lends itself to a robust process by which oversight of the practice, namely through an Ongoing Professional Practice Evaluations (OPPE) system, assures the high-quality care provided by the CP.

Some of the accomplishments the VHA has seen when incorporating the CPs in their advanced practice roles in ambulatory care are in the foundational areas of primary care, mental health, and pain management. There are over 1700 CPs with an SOP and prescriptive privileges in primary care. During Fiscal Year (FY) 2018, there were over 1.4 million patient care encounters with a primary care CP. The most common disease state interventions documented by these CPs were for diabetes management (876,154), anticoagulation (404,572), hypertension (242,607), lipid management (119,122), and tobacco cessation (60,020). In the mental health practice area there are over 360 CPs with an SOP and prescriptive privileges. During FY 2018, there were 325,358 patient care encounters with a mental health CP. These pharmacists treat a wide variety of mental health conditions, such as depression, post-traumatic stress disorder (PTSD), anxiety, insomnia, schizophrenia, bipolar disorder and substance use disorder. Additionally, VHA graduates over 600 pharmacy residents annually, of which 75 have specialized postgraduate year 2 (PGY2) mental health pharmacy residency training. In pain management, there are over 200 CPs with an SOP and prescriptive privileges. In FY 2018, those CPs provided 160,817 patient care encounters in pain management. Services provided include opioid and non-opioid medication management, high risk assessments, urine drug screening interpretation, opioid education and naloxone distribution, and non-pharmacological interventions. Applying this framework to each practice setting where CP provide direct patient care allows VHA to fully describe the roles, responsibilities, interventions and CMM services provided by the CP provider and sets the stage for practice advancement within the care setting as well as further analysis of patient care outcomes.

## Conclusions

Schwartzberg and colleagues report on clinical and other specialty services offered by pharmacists in the community in Israel and in the international arena. They rightly point out that several other countries and health systems have capitalized on the education, training and skills of the CPs in a way not yet seen in Israel or other locations. The work of CPs in the VA is an excellent example of what can be achieved when deploying these well-trained professionals broadly across the healthcare system, especially in practice areas that are pharmacotherapy intensive.

This provider model, in which a healthcare team including the pharmacist, physician, and nurse work together to form a strategic partnership focused on optimizing patient outcomes, is a crucial component to ensuring patients receive the care and services that they so greatly need.

## Data Availability

The datasets used and/or analyzed during the current study are available from the corresponding author on reasonable request.

## Abbreviations

ACPE: Accreditation Council for Pharmacy Education
APP: Advanced Practice Provider
CMM: Chronic Medication Management
CP: Clinical Pharmacists
OPPE: Ongoing Professional Practice Evaluations
Pharm. D.: Doctor of Pharmacy Degree
SOP: Scope of Practice (SOP)
VA: Veterans Administration
VHA: Veterans Health Administration
